# Interleukin 10 and Heart Fatty Acid-Binding Protein as Early Outcome Predictors in Patients With Traumatic Brain Injury

**DOI:** 10.3389/fneur.2020.00376

**Published:** 2020-06-02

**Authors:** Linnéa Lagerstedt, Leire Azurmendi, Olli Tenovuo, Ari J. Katila, Riikka S. K. Takala, Kaj Blennow, Virginia F. J. Newcombe, Henna-Riikka Maanpää, Jussi Tallus, Iftakher Hossain, Mark van Gils, David K. Menon, Peter J. Hutchinson, Henrik Zetterberg, Jussi P. Posti, Jean-Charles Sanchez

**Affiliations:** ^1^Department of Specialities of Internal Medicine, Faculty of Medicine, University of Geneva, Geneva, Switzerland; ^2^Turku Brain Injury Centre, Turku University Hospital, Turku, Finland; ^3^Department of Clinical Neurosciences, University of Turku, Turku, Finland; ^4^Perioperative Services, Intensive Care Medicine and Pain Management, Turku University Hospital and University of Turku, Turku, Finland; ^5^Department of Psychiatry and Neurochemistry, Institute of Neuroscience and Physiology, Sahlgrenska Academy at the University of Gothenburg, Mölndal, Sweden; ^6^Division of Anaesthesia, Department of Medicine, University of Cambridge, Addenbrooke's Hospital, Cambridge, United Kingdom; ^7^Division of Clinical Neurosciences, Department of Neurosurgery, Turku University Hospital Turku, Turku, Finland; ^8^Knowledge Intensive Products and Services, VTT Technical Research Centre of Finland Ltd, Tampere, Finland; ^9^Division of Neurosurgery, Department of Clinical Neurosciences, University of Cambridge, Addenbrooke's Hospital, Cambridge, United Kingdom; ^10^National Institute for Health Research, Cambridge BRC, Cambridge, United Kingdom; ^11^Royal College of Surgeons of England, London, United Kingdom; ^12^UK Dementia Research Institute at UCL, London, United Kingdom; ^13^Department of Neurodegenerative Disease, UCL Institute of Neurology, London, United Kingdom

**Keywords:** biomarker, heart fatty-acid binding protein, interleukin 10, panel, protein, outcome, traumatic brain injury

## Abstract

**Background:** Patients with traumatic brain injury (TBI) exhibit a variable and unpredictable outcome. The proteins interleukin 10 (IL-10) and heart fatty acid-binding protein (H-FABP) have shown predictive values for the presence of intracranial lesions.

**Aim:** To evaluate the individual and combined outcome prediction ability of IL-10 and H-FABP, and to compare them to the more studied proteins S100β, glial fibrillary acidic protein (GFAP), and neurofilament light (NF-L), both with and without clinical predictors.

**Methods:** Blood samples from patients with acute TBI (all severities) were collected <24 h post trauma. The outcome was measured >6 months post injury using the Glasgow Outcome Scale Extended (GOSE) score, dichotomizing patients into: (i) those with favorable (GOSE≥5)/unfavorable outcome (GOSE ≤ 4) and complete (GOSE = 8)/incomplete (GOSE ≤ 7) recovery, and (ii) patients with mild TBI (mTBI) and patients with TBIs of all severities.

**Results:** When sensitivity was set at 95–100%, the proteins' individual specificities remained low. H-FABP showed the best specificity (%) and sensitivity (100%) in predicting complete recovery in patients with mTBI. IL-10 had the best specificity (50%) and sensitivity (96%) in identifying patients with favorable outcome in patients with TBIs of all severities. When individual proteins were combined with clinical parameters, a model including H-FABP, NF-L, and ISS yielded a specificity of 56% and a sensitivity of 96% in predicting complete recovery in patients with mTBI. In predicting favorable outcome, a model consisting IL-10, age, and TBI severity reached a specificity of 80% and a sensitivity of 96% in patients with TBIs of all severities.

**Conclusion:** Combining novel TBI biomarkers H-FABP and IL-10 with GFAP, NF-L and S100β and clinical parameters improves outcome prediction models in TBI.

## Introduction

Patients with TBI may suffer from different levels and persistence of cognitive, behavioral, emotional, and physical impairments (1, 2). These impairments are frequent in patients with moderate and severe TBI, while, in cases of mild TBI (mTBI), most of the patients recover within weeks to months after the injury. However, a significant subgroup of patients with mTBI shows incomplete recovery (3–5). Despite these post-traumatic symptoms, structural brain damage in this population is often not seen using current clinical imaging modalities. The problem is significant worldwide, as mTBI accounts for 80–90% of all cases with TBI (6).

Different blood-based biomarkers have been suggested as outcome predictors of TBI to improve clinicians' abilities to optimize clinical care. Among the most studied biomarkers are the astroglial proteins S100 calcium-binding protein B (S100β), glial fibrillary acidic protein (GFAP), and the axonal protein neurofilament light (NF-L) (7–13). Other proteins, such as the anti-inflammatory protein interleukin 10 (IL-10) and the brain injury marker heart fatty acid-binding protein (H-FABP), have recently gained in interest as diagnostic tools for TBI, but little is known about their abilities as outcome predictors (14, 15).

To date, most biomarker studies have investigated proteins' individual prediction abilities. Considering the pathophysiological complexity of TBI, single biomarkers tend to have low prediction capacities, and they may therefore not be optimal for clinical use (16). To improve the accuracy, combining several biomarkers together or combining biomarker(s) with clinical parameters have been suggested, thus producing models of several predictive markers (16, 17). These kinds of models have already shown to significantly increase the predictive power compared to single markers in different diseases, such as aneurysmal subarachnoid hemorrhage, lung cancer, and sleeping sickness (18–21). Recent studies have also highlighted the beneficial use of panels as diagnostic tools for mTBI (22, 23) and for predicting the need for acute head imaging following TBI (24). Furthermore, combining inflammatory proteins together with brain-derived ones may improve the ability to predict outcome after TBI (25, 26).

The aim of the present study was to compare the proteins IL-10 and H-FABP to the well-studied proteins S100β, GFAP, and NF-L for their individual ability to predict patients who will have favorable long-term outcome analyzing patients on a clinically meaningful allocation basis: patients with mTBI and patients with TBIs of all severities. In order to demonstrate the added value of blood biomarkers in addition to clinical variables, we analyzed the biomarkers in isolation and with clinical variables recorded upon admission. Furthermore, combinations of predictive markers were evaluated in an attempt to increase this prediction ability.

## Materials and Methods

### Study Population and Clinical Variables

In this single-center study, patients were recruited at Turku University Hospital (a tertiary care university hospital with a combined primary and tertiary care emergency department) in Finland between the years 2011–2013. All the consecutive patients with TBI were evaluated for eligibility to be recruited in the study by the research team between 8 a.m. to 10 p.m. To be included in the study, the following inclusion criteria needed to be fulfilled; age ≥18 years, hospital admission within 24 h after trauma, clinical diagnosis of a TBI with an indication for a head computed tomography (CT) scan according to the NICE criteria (27) as judged by an emergency physician on call, and outcome data at 6–12 months after injury had to be available. Exclusion criteria were penetrating or blast-induced injury, chronic subdural hematoma, inability to live independently due to a previous brain disease, no performed CT scan, or no written consent.

The ethical review board of Hospital District of South-West Finland approved the study protocol (decision 68/180/2011). Written informed consent was obtained from all participants or their legal representatives prior to inclusion.

The outcomes were assessed by single experienced neurologist (OT) using the Glasgow Outcome Scale Extended (GOSE) (28). The total injury burden was assessed with Injury Severity Score (ISS) (29).

### Head Imaging and Traumatic Brain Injury Severity Classifications

Head CT scans were classified according to the Marshall classification system (30). Neuroradiologists at the Turku University Hospital and a senior neurosurgeon (JPP) double-read the CT scans.

In addition to using the lowest Glasgow Coma Scale (GCS) score before possible intubation, either at the scene of accident or emergency department (31), TBI severity was also classified using an aggregate covariate combining the lowest GCS and the length of post-traumatic amnesia^1^.

### Protein Measurement

Serum samples were drawn within 24 h after trauma. However, as these different time points did not appear to correlate with biomarker levels, all of them were considered as a common time point. Only admission samples were assessed. After obtaining the blood samples, the samples were centrifuged and stored at −70 °C. The proteins GFAP and NF-L were measured using the Human Neurology 4-plex A assay (N4PA) on HD-1 single molecule array (Simoa) device from Quanterix (Lexington, MA, USA). The lower limit of quantification (LLoQ) for each kit was 0.104 pg/mL for NF-L and 0.221 pg/mL for GFAP. The protein S100β was measured using the EZHS100B-33K kit from Millipore (Millipore, Billerica, MA, USA) with an LloQ of 2.74 pg/mL. H-FABP and IL-10 were analyzed using the K151HTD and K151QUD kits, respectively, Meso Scale (Meso Scale Diagnostics, Rockville, MD, USA). The LloQ for H-FABP was 0.137 ng/mL and for IL-10 0.298 pg/mL. All proteins were measured according to manufacturers' recommendations by board-certified laboratory technicians who were blinded to clinical data.

### Statistical Analysis

Statistical analyses were performed to evaluate the proteins' outcome prediction ability defined by the GOSE score. The patients were dichotomized in two different groups: (a) the first one included TBI patients of all severities (worst GCS 3–15, *n* = 88), while the second one (b) included only the subgroup of mTBI (worst GCS 13–15, *n* = 49) patients. The GOSE was dichotomized into (a) 1–4 for unfavorable outcome or 5–8 for favorable outcomes for all severity patients and (b) 1–7 for incomplete recovery or 8 for complete recovery for mTBI patients. These dichotomizations were chosen because the favorable/unfavorable outcome is relevant for TBIs in general (especially moderate—severe TBIs) and complete/incomplete for those with mTBI. Non-parametric tests were used because all proteins were non-normally distributed, as indicated by the Kolmogorov-Smirnov test (*p* < 0.05). Therefore, to evaluate the proteins' differences between groups, the Mann-Whitney U test was performed using IBM SPSS software, version 24.0 (SPSS Inc., Chicago, IL, USA). The proteins' outcome prediction capacities were evaluated using the partial area under a receiver operating characteristic cure (pAUC), which allowed us to focus only in the interest region of the ROC curve. Analysis were computed using TIBCO Spotfire S+® version 8.2 software (TIBCO Software Inc., Palo Alto, CA, USA). Model selections were done using the PanelomiX toolbox based on the iterative combination of biomarkers/variables and thresholds method (ICBT) (32). To make the process faster, several biomarkers (H-FABP, IL-10, S100β, NF-L, and GFAP)/variables (age, Marshall grade, Injury Severity Score, Severity, and GCS) were tested together. PanelomiX used the random forest method to select the different thresholds. Cross validation and ROC analysis were used to evaluate the performance of the model. In this manuscript, a maximum number of three biomarkers or clinical parameters in each model were investigated. The PanelomiX tool established the best cut-off for each single biomarker, and the best combinations were investigated when the sensitivity was set at 95–100% in order to reduce the false negative cases.

## Results

### Study Population

A total of 88 TBI patients with blood samples available within 24 from the time of injury were included in this study, and 69% of the patients were male. The most common causes of trauma were falls and traffic accidents. The distributions of severity, imaging findings, and outcome are shown in Table 1.

**Table 1:** Characteristics of the study patients.

	**TBI all severities**				**Mild TBI**			
	**Complete recovery**		**Favorable outcome**		**Complete recovery**		**Favorable outcome**	
	**GOSE 8**	**GOSE 1–7**	**GOSE 5–8**	**GOSE 1–4**	**GOSE 8**	**GOSE 1–7**	**GOSE 5–8**	**GOSE 1–4**
	**(*n* = 25)**	**(*n* = 63)**	**(*n* = 60)**	**(*n* = 28)**	**(*n* = 25)**	**(*n* = 24)**	**(*n* = 45)**	**(*n* = 4)**
**Age**								
Mean (SD)	40.9 (20.27)	51.13 (18.39)	44 (18.68)	57.25 (18.02)	40.9 (20.3)	47.5 (18.7)	43.4 (19.6)	52.8 (20.9)
**Gender**								
Male, n (%)	17 (68)	44 (69.8)	43 (71.7)	18 (64.3)	17 (68)	9 (37.5)	31 (68.9)	1 (25)
**Marshall Grade**, ***n*** **(%)**								
No visual pathology (grade 1)	17 (68)	18 (28.6)	33 (55)	2 (7.1)	17 (68)	11 (45.8)	28 (62.3)	0 (0)
Diffuse injury (grade 2)	4 (16)	11 (17.5)	11 (18.3)	4 (14.3)	4 (16)	9 (37.5)	10 (22.2)	3 (75)
Diffuse injury with swelling (grade 3)	0 (0)	1 (1.6)	0 (0)	1 (3.6)	0 (0)	0 (0)	0 (0)	0 (0)
Diffuse injury with shift (grade 4)	0 (0)	0 (0)	0 (0)	0 (0)	0 (0)	0 (0)	0 (0)	0 (0)
Evacuated mass lesion (grade 5)	1 (4)	22 (34.9)	9 (15)	14 (50)	1 (4)	1 (4.2)	2 (4.4)	0 (0)
Non evacuated mass lesion (Grade 6)	3 (12)	11 (17.5)	7 (11.7)	7 (25)	3 (12)	3 (12.5)	5 (11.1)	1 (25)
**Injury Severity Score (ISS)**, ***n*** **(%)**								
Minor 1–8	16 (64)	13 (20.6)	27 (45)	2 (7.1)	16 (64)	10 (41.7)	25 (55.6)	1 (25)
Moderate 9–15	4 (16)	15 (23.8)	13 (21.6)	6 (21.4)	4 (16)	8 (33.3)	10 (22.2)	2 (50)
Serious 16–24	4 (16)	16 (25.3)	12 (20)	8 (28.6)	4 (16)	4 (16.7)	7 (15.6)	1 (25)
Severe 25–49	1 (4)	17 (26.9)	7 (11.7)	11 (39.3)	1 (4)	2 (8.3)	3 (6.7)	0 (0)
Critical 50–74	0 (0)	1 (1.6)	1 (1.7)	0 (0)	0 (0)	0 (0)	0 (0)	0 (0)
Maximum 75	0 (0)	1 (1.6)	0 (0)	1 (3.6)	0 (0)	0 (0)	0 (0)	0 (0)
*Severity, ***n*** **(%)**								
Very mild 1	1 (4)	0 (0)	1 (1.7)	0 (0)	1 (4)	0 (0)	1 (2.2)	0 (0)
Mild 2	23 (92)	27 (42.9)	45 (75)	5 (17.9)	23 (92)	18 (75)	39 (8.7)	2 (50)
Moderate 3	0 (0)	12 (19.1)	7 (11.7)	5 (17.9)	0 (0)	4 (16.6)	3 (6.7)	1 (25)
Severe 4	1 (4)	10 (15.9)	5 (8.2)	6 (21.4)	1 (4)	1 (4.2)	1 (2.2)	1 (25)
Very severe 5	0 (0)	12 (19.1)	1 (1.7)	11 (39.3)	0 (0)	0 (0)	0 (0)	0 (0)
Unknown	-	2 (3.1)	1 (1.7)	1 (3.6)	0 (0)	1 (4.2)	1 (2.2)	0 (0)
**GCS**, ***n*** **(%)**								
Mild 13–15	25 (100)	24 (38.1)	45 (75)	4 (14.2)	25 (100)	24 (100)	45 (100)	4 (100)
Moderate 9–12	0 (0)	24 (38.1)	12 (20)	12 (42.9)	0 (0)	0 (0)	0 (0)	0 (0)
Severe 3–8	0 (0)	15 (23.8)	3 (5)	12 (42.9)	0 (0)	0 (0)	0 (0)	0 (0)
**Time from injury to blood sampling**								
Mean, hours (SD)	6.2 (5.7)	9.9 (6.1)	9.8 (6.1)	12.3 (6.6)	6.2 (4.8)	12.9 (5.7)	9 (10.3)	14 (4.6)

* *Severity combined from GCS and duration of posttraumatic amnesia, see ref ^1^*.

### Complete Recovery – Patients With mTBI

We first investigated the proteins' individual capacities to predict complete recovery in patients with mTBI. All these proteins tended to be higher in patients with incomplete recovery (GOSE ≤ 7, *n* = 24) compared to those with complete recovery (GOSE 8, *n* = 25), although these findings were without significance. The proteins were investigated when the sensitivity was set at 95–100%. The individual specificities remained low. The best performing protein was H-FABP, reaching 4% specificity and 100% sensitivity. NF-L and S100β had somewhat higher specificities of 12% but lower sensitivities of 96% (Table 2).

**Table 2:** Performance of single proteins when differentiating between complete (GOSE 8) and incomplete (GOSE ≤ 7) recovery in patients with mTBI.

	**% pAUC (95% CI)**	**Threshold**	**%SP (95% CI)**	**95–100 %SE (95% CI)**
H-FABP	0.2 (0.0–0.7)	44.9	4.0 (0.0–12)	100 (100–100)
NF-L	0.1 (0.0–1.2)	4.85	12 (0.0–24.1)	95.8 (87.5–100)
S100β	0.1 (0.0–1.0)	23.17	12 (0.0–28.0)	95.8 (87.5–100)
IL-10	0.0 (0.0–0.7)	–	0.0 (0–0)	100 (100–100)
GFAP	0.0 (0.0–0.6)	–	0.0 (0–0)	100 (100–100)

*Biomarkers are shown according to their specificity obtained at 95–100% sensitivity. pAUC, partial area under the curve; SP, specificity; SE, sensitivity; IL-10, interleukin 10; H-FABP, heart fatty-acid binding protein; GFAP, glial fibrillary acidic protein; NF-L, neurofilament light; S100β, S100 calcium-binding protein B. All threshold concentrations are in pg/mL except for H-FABP which is in ng/mL*.

Next, combinations of proteins to increase the ability to predict complete recovery were evaluated. The best two-protein panel was a combination of H-FABP and NF-L, reaching a specificity of 40% and sensitivity of 96%, thus resulting in a 36-percentage point increase in specificity compared to the best performing single protein H-FABP. A panel comprising three proteins—H-FABP, GFAP, and NF-L—was further capable of increasing the specificity to 44% with 96% sensitivity (Table 3).

**Table 3:** Panels performed in patients with mTBI, including proteins (H-FABP, IL-10, GFAP, S100β, and NF-L) or proteins and clinical parameters: Marshall grade, severity, injury severity score, and age.

**Panel**	**Markers cut-off**	**n GOSE 8**	**n GOSE <7**	**% SP (95% CI)**	**% SE (95% CI)**
2 Parameters (only proteins)	H-FABP (6.26)	25	24	40.0 (20.0–60.0)	95.8 (87.5–100)
	NF-L (15.9)				
3 Parameters (only proteins)	H-FABP (5.01)	25	24	44.0 (24.0–64.0)	95.8 (87.5–100)
	NF-L (13.46)				
	GFAP (2457.5)				
2 Parameters (proteins/clinical parameters)	H-FABP (6.26)	25	24	40.0 (20.0–60.0)	95.7 (87.0–100)
	NF-L (15.9)				
3 Parameters (proteins/clinical parameters)	H-FABP (4.30)	25	24	56.0 (36.0–76.0)	95.7 (87.0–100)
	NF-L (13.46)				
	Severity (2.5)				

*Complete recovery GOSE 8 and incomplete recovery GOSE ≤ 7; SP, specificity; SE, sensitivity. All protein cut-off concentrations are in pg/mL except for H-FABP, which is in ng/mL*.

Combining individual proteins with clinical parameters was also evaluated to predict complete recovery (Table 3). Combination of H-FABP, the most specific molecule in isolation, with clinical parameters showed an improved performance over proteins alone. Maintaining a value of 96% sensitivity, the combination of H-FABP, NF-L, and TBI severity reached a specificity value of 56%, which is far better than for H-FABP alone (Table 3). The individual outcome prediction capacities of clinical parameters are presented in Supplementary Table 1.

### Favorable Outcome – Patients With TBIs of All Severities

H-FABP, IL-10, S100β, NF-L, and GFAP were evaluated for their individual capacities to predict favorable outcome in patients with TBIs of all severities. The blood levels of all proteins studied were significantly higher in patients with unfavorable outcome (GOSE ≤ 4, *n* = 28) compared to those with favorable outcome (GOSE ≥ 5, *n* = 60) (*p* ≤ 0.001). Again, the ability of each biomarker for the detection of favorable outcome was evaluated when the protein reached 95–100% sensitivity. According to these criteria, the best performing protein was IL-10, with a sensitivity reaching 96% and specificity of 50%. The proteins H-FABP and GFAP performed similarly, with sensitivities of 96% for both and specificities of 30% and 28%, respectively (Table 4).

**Table 4:** Performance of single proteins when differentiating between favorable (GOSE 5–8) and unfavorable (GOSE 1–4) outcomes in all severity patients.

	**% pAUC (95% CI)**	**Threshold**	**%SP (95% CI)**	**95-100 %SE (95% CI)**
IL-10	1.4 (0.7–3.0)	0.39	50.0 (36.7–63.3)	96.4 (89.3–100)
H-FABP	1.1 (0.6–2.7)	4.31	30.0 (18.3–41.7)	96.4 (89.3–100)
GFAP	0.8 (0.3–2.3)	415	28.3 (16.7–40.0)	96.4 (89.3–100)
NFL	0.5 (0.2–4.4)	5.47	10.0 (3.3–18.3)	100 (100–100)
S100β	0.1 (0–1.4)	23.17	6.7 (1.7–13.3)	96.4 (89.3–100)

*Biomarkers are shown in order according to their specificity obtained at 95–100% sensitivity. Mann U, Mann-Whitney U-test; pAUC, partial area under the curve; SP, specificity; SE, sensitivity; IL-10, interleukin 10; H-FABP, heart fatty-acid binding protein; GFAP, glial fibrillary acidic protein; NF-L, neurofilament light; S100β, S100 calcium-binding protein B. All threshold concentrations are in pg/mL except for H-FABP, which is in ng/mL*.

The proteins' performances were also evaluated when they were combined in panels and when they were combined with clinical parameters. Individual performances of clinical parameters are presented in Supplementary Table 2.

The best combination using two proteins was IL-10 and H-FABP. This panel was capable of reaching 96% sensitivity and 58% specificity. This combination increased the specificity with 8 percentage points compared to the best single molecule IL-10. The best performing panel combining three proteins included IL-10, H-FABP, and GFAP. This panel managed to maintain the sensitivity at 96% and to increase the specificity up to 63% (Table 5).

**Table 5:** Panels including only proteins (H-FABP, IL-10, GFAP, S100β, and NF-L) or proteins and clinical parameters (Marshall grade, severity, injury severity score, age, and GCS).

**Panel**	**Markers cut-off**	**n GOSE≥5**	**n GOSE≤4**	**% SP (95% CI)**	**% SE (95% CI)**
2 Parameters (only proteins)	IL-10 (0.38)	60	28	58.3 (45.0–71.7)	96.4 (89.3–100)
	H-FABP (4.31)				
3 Parameters (only proteins)	IL-10 (0.38)	60	28	63.3 (51.7–75.0)	96.4 (89.3–100)
	H-FABP (4.31)				
	GFAP (145.13)				
2 Parameters (proteins/clinical parameters)	NF-L (41.5)	59	27	72.9 (61–84.7)	96.3 (88.9–100)
	Severity (2.5)				
3 Parameters (proteins/clinical parameters)	IL-10 (0.44)	59	27	79.7 (69.5–89.8)	96.3 (88.9–100)
	Age (61)				
	Severity (2.5)				

*Favorable outcome GOSE ≥5 and unfavorable outcome GOSE ≤ 4; SP, specificity; SE, sensitivity. All protein cut-off concentrations are in pg/mL except for H-FABP, which is in ng/mL*.

The combination of individual proteins with clinical parameters improved the predictive ability compared to predictions using only protein biomarkers. Combining IL-10, the most specific protein, with patient's age and TBI severity reached 80% specificity with 96% sensitivity, which produces an increase in the specificity of 30 percentage points when comparing with the best single protein marker (Table 5).

## Discussion

Reliable early prediction of the patient's outcome in TBI can help clinicians in their decision-making and thereby optimize the care cost-effectively. Different blood biomarkers have previously been suggested as objective outcome predictor tools. This prospective, observational study of patients with acute TBI showed the potential benefits of using novel TBI biomarkers IL-10 and H-FABP but also previously studied biomarkers GFAP (13, 33), S100β (34), and NF-L (11, 33, 35) as individual predictive biomarkers for promising outcome prediction; this also showed that models including previously known robust clinical predictors may further improve the accuracy.

According to the results presented in this manuscript we can highlight that blood biomarkers have a strong outcome-predictive capacity in patients with TBI and more importantly that the combination with other biomarkers or clinical parameters enhances this precision.

The present study has shown that, in mTBI patients, among all the tested proteins, H-FABP exhibits the best capacity in discriminating patients with complete and incomplete recovery. This protein was also selected among the most promising ones when the predictive capacity of panel of proteins was evaluated; the combination of GFAP, H-FABP, and NF-L reached a specificity value of 40% when the sensitivity was set at 95–100%. Furthermore, as previously stated, inclusion of different clinical covariates showed to importantly augment the outcome prediction capacity of the proteins. Models including H-FABP, NF-L, and ISS yielded a specificity of 56%

In regard to patients with TBIs of all severities, IL-10 has the best capacity to discriminate favorable and unfavorable outcome; however, similarly to the mTBI population, the specificities of all proteins studied remained ≤ 50% when studied in isolation. Based on these findings, we again studied the proteins in panels fixing the sensitivity at 95–100%, which allowed us to find that the combination of GFAP, H-FABP, and IL-10 reached a specificity value of 63%. Once again, the inclusion of clinical covariates drastically improved the results reaching a specificity of 80% when IL-10, age and TBI were combined together.

Briefly, the current results indicate that of all studied proteins H-FABP has the best capacity to predict complete recovery and IL-10 to predict favorable outcome. In all two- and three-biomarker panels, either H-FABP and IL-10 were included. These markers have previously been shown efficient as CT scan triage tool for patients with mild TBI (22).

The protein H-FABP is a small cytoplasmic protein that leaks out from injured endothelial cells and neuronal cell bodies. It is a well-known myocardial infarction biomarker, and, even if it is not a TBI specific marker, increased levels have previously been shown in patients with severe TBI and poor outcome (36–39). IL-10 or human cytokine synthesis inhibitory factor is a potent anti-inflammatory cytokine mainly produced by macrophages, B cells, and dendritic cells. Similarly, it is not a brain-specific marker, but the levels have also been shown to be increased in patients with severe TBI and poor outcome (40). Literature on the prognostic value of both H-FABP and IL-10 is scarce. The previous reports on the outcome prediction ability of IL-10 after TBI are contradictory, though it has shown some potential in the prediction of mortality—the past studies have been very heterogenic in their methods (41). Intriguingly, the best-performing three-biomarker panel in discriminating patients with favorable and unfavorable outcome in patients with TBIs of all severities (GFAP, H-FABP, and IL-10) was the same that we have previously reported to be the best panel in detecting patients with CT-positive findings in the groups of patients with TBIs of all severities and patients with mTBIs without extracranial injuries (24). Because all these studies are from the same patient population, further studies should confirm if these combinations perform best also in other patients with acute TBI.

Several different pathophysiological processes can affect the outcome of TBI, such as apoptosis, blood brain barrier damage, and neuroinflammation (42). TBI is a complex pathophysiological disease where single markers may never be accurate enough for clinical applications (43). Combining biomarkers from different pathophysiological pathways has previously been shown to increase the overall accuracy (16, 24). Among other clinical parameters, such as age, Marshall grade, or ISS, we chose to include both the GCS score and TBI severity (an aggregate covariate) to the predictive models consisting of clinical covariates and biomarkers because we hypothesized that it would better reflect the importance of PTA in combination with the GCS score. As seen in the results, TBI severity is included more often in the predictive models than the GCS score alone. The prediction capacity of biomarkers is usually evaluated using the total AUC value; however, this entire value of ROC curve evaluates regions that usually are not relevant to clinical applications. Therefore, in order to avoid this drawback we decided to focus on pAUC, evaluating specificity values only when the sensitivity was fixed between 95 and 100% (44).

The model selection method used in this manuscript, PanelomiX, has been shown to have several advantages over other already known selection methods, such as random forest, support vector machine or logistic regression. It has provided us the selection of best biomarkers specifying their optimal cut-off points (obtained by cross-validation). Obtained results are easy to interpret for the clinicians, which is another important advantage over other traditional black-box methods, and furthermore the prediction performances of panels obtained using PanelomiX tool are similar to that obtained with support vector machine among others.

This study has several limitations. Blood samples were collected within the first 24 h after trauma, which could be too late for the measurement of several biomarkers as S100β and GFAP (24, 45). The optimal biomarker panel is probably greatly dependent on both the time from injury and type of injury, and this is why further studies are necessary to clarify which biomarkers or biomarker combinations perform best at different points of time in different TBI populations. There is also some evidence that the clinical usability of biomarkers may depend on the age of the patient (46). Furthermore, the cohort used here included only 88 patients with TBI, making multivariate analysis of the molecules inconvenient according to the Monte Carlo study (47). The fairly small study population also increases the risk of over-fitting bias, which is why the results should be verified and validated in a larger cohort. Our study cohort included patients with TBI of all the severities where 56% had mTBI and 17% had severe TBI. For the whole cohort, the results may be partly driven by the more severe cases of TBI due to their higher biomarker levels, and, on the other hand, our cases of mTBI were more severe than patients with mTBI as a whole since most of our cases had been admitted to neurosurgical ward. We chose to study patients with mTBI and TBIs of all severities independently because (i) patients with mTBI are a distinctive group of patients with specific diagnostic needs, and (ii) examining the outcome prediction ability in patients with all severities is important because initial severity of a TBI is not always clear due to possible confounding factors (such as intoxication, hypoglycemia, and neurological diseases).

Moreover, using GOSE as an outcome measure is a relatively insensitive way to detect subtle impairments in patients with mTBI or in TBI in general since it will miss many important and subtle deficits and it may be affected also by factors not directly related to the anatomical brain injury itself. The usability of IL-10 and H-FABP should be studied using a higher number of patients with different clinical characteristics.

## Conclusion

The novel proteins IL-10 and H-FABP in TBI diagnostics show promise in detecting patients with either favorable outcome or complete recovery following TBI. Combining levels of these proteins and NF-L with clinical covariates may assist in clinical decision-making at the emergency department and stratification for different monitoring and treatment algorithms.

## Data Availability Statement

The datasets analyzed in this article are not publicly available. Requests to access the datasets should be directed to leire.azurmendi@unige.ch.

## Ethics Statement

The studies involving human participants were reviewed and approved by 68/180/2011. The patients/participants provided their written informed consent to participate in this study.

## Author Contributions

LL, LA, and J-CS conceived and designed the study with critical contributions from OT and JP. OT, JP, AK, RT, H-RM, and JT recruited the patients and collected the data with critical contributions from IH. LL, LA, J-CS, HZ, and KB were responsible for analytical biomarker assessments. LL, LA, and J-CS conducted the statistical analyses. LL, LA, OT, JP, and J-CS drafted the manuscript. All authors substantially contributed to the revision of the manuscript. OT, VN, MG, PH, JP, and J-CS supervised the study. JP and J-CS take the responsibility for the paper as whole.

## Conflict of Interest

The authors declare that the research was conducted in the absence of any commercial or financial relationships that could be construed as a potential conflict of interest.

## Footnotes

^1^https://www.healthquality.va.gov/guidelines/Rehab/mtbi/mTBICPGFullCPG50821816.pdf
