# Supplementary material for: Interleukin 10 and Heart Fatty Acid-Binding Protein as Early Outcome Predictors in Patients With Traumatic Brain Injury
Source: Front Neurol. 2020 Jun 2;11:376. doi: 10.3389/fneur.2020.00376 (PMC7280446; doi:10.3389/fneur.2020.00376)
Supplement: Supplementary file 1 [file Data_Sheet_1.docx]

**Supplementary table 1. Clinical parameters performances to differentiate between complete (GOSE 8) and incomplete (GOSE ≤ 7) recovery patients in patients with mTBI.**

|  | **% pAUC (95% CI)** | **Threshold** | **%SP (95% CI)** | **95-100 %SE (95% CI)** |
| --- | --- | --- | --- | --- |
| **GCS** | 0.4 (0.1-0.9) | 13.5 | 4.0 (0-0-12.0) | 100 (100-100) |
| **Severity** | 0.3 (0.1-0.8) | 1.5 | 4.0 (0.0-12.0) | 100 (100-100) |
| **Marshall grade** | 0.2 (0.1-0.3) | - | 0.0 (0-0) | 100 (100-100) |
| **Age** | 0.0 (0.0-2.0) | - | 0.0 (0-0) | 100 (100-100) |
| **ISS** | 0.0 (0.0-1.2) | - | 0.0 (0-0) | 100 (100-100) |

SP: specificity; SE: sensitivity. ISS: injury severity score. Severity was classified using an aggregate covariate combining the lowest Glasgow Coma Scale (GCS) before possible intubation and the length of posttraumatic amnesia.

**Supplementary table 2. Clinical parameters performances to differentiate between favorable (GOSE 5-8) and unfavorable (GOSE 1-4) outcome in all severity patients.**

|  | **% pAUC (95% CI)** | **Threshold** | **%SP (95% CI)** | **95-100 %SE (95% CI)** |
| --- | --- | --- | --- | --- |
| **Marshall grade** | 0.9 (0.4-3.2) | - | 0 (0-0) | 100 (100-100) |
| **Severity** | 0.6 (0.3-1.6) | 1.5 | 1.7 (0-5.1) | 100 (100-100) |
| **ISS** | 0.6 (0.0-2.8) | 5.5 | 36.7 (25-50) | 96.4 (89.3-100) |
| **GCS** | 0.4 (0.2-1.2) | - | 0 (0-0) | 100 (100-100) |
| **Age** | 0.3 (0.1-1.9) | 20.5 | 8.3 (1.7-16.7) | 96.4 (89.3-100) |

SP: specificity; SE: sensitivity. ISS: injury severity score. Severity was classified using an aggregate covariate combining the lowest Glasgow Coma Scale (GCS) before possible intubation and the length of posttraumatic amnesia
